# A Study on the Correlation between Change in the Geometrical Dimension of a Free-Falling Molten Glass Gob and Its Viscosity

**DOI:** 10.3390/s22020661

**Published:** 2022-01-15

**Authors:** Mazhar Hussain, Mattias O’Nils, Jan Lundgren, Irida Shallari

**Affiliations:** STC Research Centre, Mid-Sweden University, Holmgatan 10, 85170 Sundsvall, Sweden; Mattias.ONils@miun.se (M.O.); Jan.Lundgren@miun.se (J.L.); Irida.Shallari@miun.se (I.S.)

**Keywords:** glass melt, glass gob, glass containers, glass plant, image processing, multi-camera, gob viscosity, sensor fusion

## Abstract

To produce flawless glass containers, continuous monitoring of the glass gob is required. It is essential to ensure production of molten glass gobs with the right shape, temperature, viscosity and weight. At present, manual monitoring is common practice in the glass container industry, which heavily depends on previous experience, operator knowledge and trial and error. This results in inconsistent measurements and consequently loss of production. In this article, a multi-camera based setup is used as a non-invasive real-time monitoring system. We have shown that under certain conditions, such as keeping the glass composition constant, it is possible to do in-line measurement of viscosity using sensor fusion to correlate the rate of geometrical change in the gob and its temperature. The correlation models presented in this article show that there is a strong correlation, i.e., 0.65, between our measurements and the projected viscosity.

## 1. Introduction

**Figure 1:**
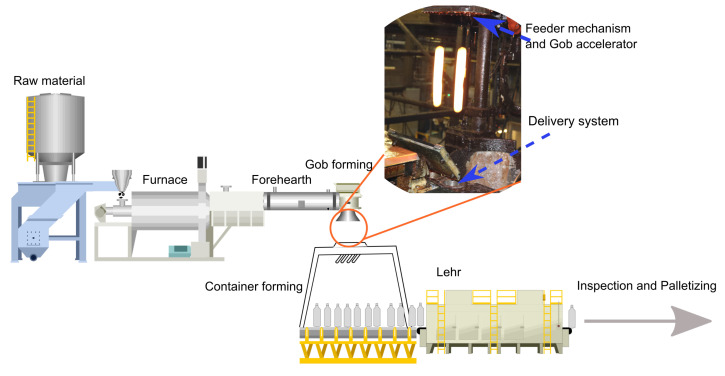
The figure shows a typical glass container manufacturing plant from hot end to cold end. The dotted circle shows the measuring point where molten gobs free-fall between the feeder mechanism and delivery system to enter into the glass forming machine [8].

There are many requirements in the glass container manufacturing industry to produce a molten glass gob that has acceptable size, shape, temperature, viscosity and weight [1,2]. Only a glass gob with acceptable characteristics would be used to form a fault free glass container. The molten glass gob is produced in a fore-hearth (feeder) [3,4] by cutting the glass into small chunks called gob. These chunks of molten glass are then dropped into a delivery system from where this gob is transported to a glass forming machine to produce glass containers [5] as shown in Figure 1. There are a number of factors that could affect the formation of a gob [6], these factors could be in the mechanical components of glass forming machines, the synchronization between different parts of the machine or any changes in the raw material such as the viscosity of the glass melt [6]. There are various methods to estimate the viscosity of the molten glass itself [7], however, most of them only work in a lab environment and require contact with the glass melt [7], which may introduce impurities and slow down the manufacturing process.

Typically, there are four primary operations in glass manufacturing, i.e., batching, melting, fining and forming. The production of molten glass gob that has a nice shape, homogeneity in temperature and viscosity with the desired mass and weight starts in the batching and melting stages [8]. However, when the molten glass is cut and dropped, with the pull of gravity from the fore-hearth, the glass gob travels in a state of free-fall for a while before it enters the delivery system that is attached to the glass container forming machines. At this stage, slight variations could compromise the quality of the final product.

**Figure 2:**
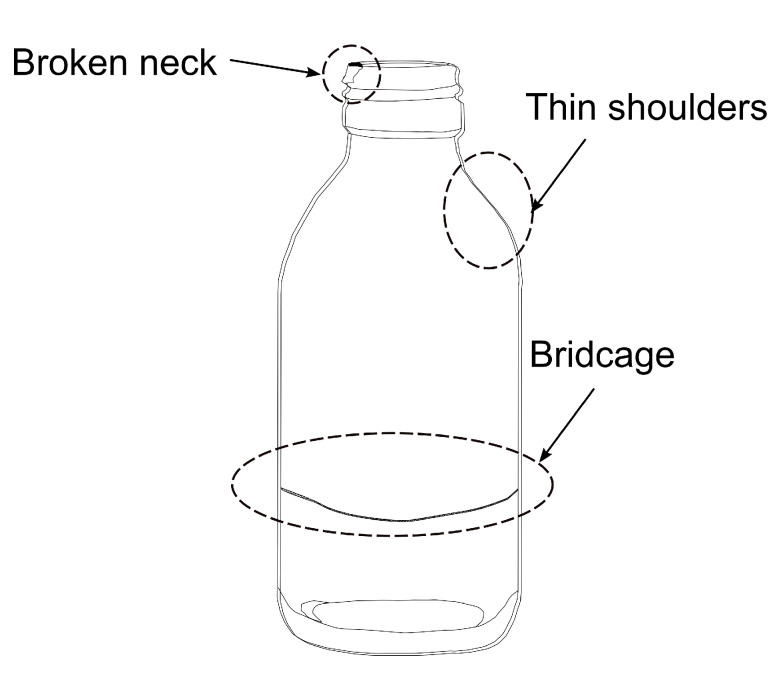
Glass container demonstrating broken neck, thin shoulders, or birdcage (a string of glass inside the container).

In the case of a glass container, the variation in viscosity could create defects such as thin shoulders, birdcage and broken neck, as shown in Figure 2.

Homogeneous viscosity throughout the glass production process is important. Slight changes in viscosity in molten glass can cause various defects in the final product. There is a lack of knowledge about the viscosity variation during this critical phase when the molten gob is in the air. Current methods to estimate the viscosity are mostly offline [9,10,11,12,13,14,15] and only used to estimate the viscosity of molten glass in the furnace. These viscosity estimates do not provide any indication regarding a prospective change in viscosity after the molten glass in the form of a gob leaves the fore-hearth and enters the glass forming machine. We know that glass viscosity has a very high dependence on temperature, and extreme free surface deformations could cause defects in the final product [16], therefore, it is of utmost importance to have continuous contactless monitoring of the molten gob to ensure the quality of the glass containers.

In our previous work [17], we presented an in situ multi-camera setup that can dynamically characterize small changes in geometrical properties of a free-falling molten object. In this article, we rely on sensor fusion [18] that consists of a multi-camera setup to measure the geometrical changes in a molten glass gob, and we model the changes with regards to its viscosity. The main contribution of this article is a novel model using the correlation of geometrical changes that can occur in the glass gob with variations in viscosity. This correlation model presents a possibility to estimate the variation in the viscosity of a molten gob based on shape analysis, as for example using the shrinkage rate of the molten gob obtained from the setup in [17]. Furthermore, we verified our correlation model with simultaneous measurements using a high-speed thermal camera, taking advantage of the linear relationship between the temperature of molten glass and its viscosity.

## 2. Related Work

In glass manufacturing, viscosity along with temperature are the most important properties [9]. Accurate viscosity estimation using reliable measurement methods results in a homogeneous glass melt for the glass forming process. There are a number of methods used to determine the viscosity of molten glass in different ranges; however, we noticed that almost all methods are either offline and require a lab to perform the measurements, or they are non-invasive, but only work for low viscous liquids and liquids with certain properties [19]. Paper [10] presents a device called rotational viscometer that is used to measure viscosities in the range of 1 to 106 Pa.s by measuring the torque of a rotating spindle immersed in molten glass in a crucible. This is an offline method and can only be used for glass melts in a furnace. The glass melt still has to travel to the fore-hearth and from there to the glass forming machines. Joshi et al. [11] introduced another method called parallel plate viscosimeter that requires samples of glass melt to perform the procedure of parallel plate viscometry to estimate the viscosity of glass melts in a lab. Methods using falling-sphere viscometry, [12,13], require similar conditions as the parallel plate method. There are various other methods such as beam-bending [14], fiber elongation [15,20], etc., that have been proven to estimate the viscosity of glass melts in different stages with good accuracy; however, they are all offline and require a laboratory setup. Other than these methods, there are some contactless methods that can be used to measure the viscosity of liquids in general. The method presented in [21] uses an ultrasonic interferometric sensor to measure the rheological properties of a glycerine–water mixture. This method is only for viscosities that are far lower than that of glass, in addition, the method requires calibration and nulling, making it impossible to use as an online tool. Another contactless method is the free-fall drop oscillation method [22] that provides an effective approach to measure viscosity in melted copper and nickel based on a high-speed camera measuring the oscillations in droplets. However, in their analysis, the droplets are very small, spherical and have an order of magnitude lower viscosity compared to the molten glass gob. Similarly, in [23], a line scan camera is used to estimate the viscosity of a free-falling water droplet. Their method relies on fast oscillation and damping, which is not the case for molten glass gobs because of the much higher viscosity.

### 2.1. Relation between the Surface Tension and the Temperature of Molten Glass

Griffith [24] did an experiment to find a relation between the surface tension of molten glass and its temperature while developing a theory regarding the phenomena of rupture and flow in solids. From his analysis he concluded that the surface tension of soda-lime-silica glass has a linear relationship with temperatures between 700 ${}^{\circ }$C to 1100 ${}^{\circ }$C. Griffith uses the Quincke drop-shape method to determine the surface tension of glass at 1100 ${}^{\circ }$C and sagging in a fiber method for lower temperatures.

### 2.2. Relation between the Surface Tension and the Viscosity of Molten Glass

Washburn et al. [25] determined the surface tension of molten glass with different compositions. They observed that as the viscosity decreased, the forces of surface tension increased and significantly influenced the flow of molten glass. This proved the relationship between surface tension and the viscosity of soda-lime-silica glasses for certain ranges. Similar results were found using “the bulb method” presented by [26].

Molten gob is produced under high temperature and with high viscosity making it behave neither as a solid, nor as a liquid; the latter is due to high surface tension. We believe that there is a linear relationship between change in gob shape due to its surface tension and viscosity. Our hypothesis in this article is that the surface tension of the molten gob affects the geometrical properties of the gob and these geometrical changes in gob can be used to estimate variations in viscosity. In order to verify this hypothesis, a literature study has been conducted to explore the relationship between the temperature and viscosity of molten glass to its surface tension. In this article, we have used the knowledge from [24,25] about the linear relationship between the surface tension and temperature of molten glass to strengthen our hypothesis about a connection between gob shape and its temperature and eventual viscosity.

## 3. Theory

### 3.1. The Importance of Glass Composition

Glass composition plays an important role in the quality of the final product, as it influences the properties of glass mold in a predictable way [7]. For example, in order to obtain the same viscosity for different glass compositions, we would have to use different temperatures. Hence, viscosity is also highly dependent on the glass composition [9].

### 3.2. Viscosity for Glass Container Manufacturing

For glass container manufacturing, there are certain viscosity ranges that are important during various steps in the production, as shown in Table 1 from [27] with viscosity in Pa.s. For example, depending on the glass composition, the viscosity range for producing gobs for container forming should be between $10^{2.6}$ Pa.s ($10^{3.6}$ Poise) to $10^{3}$ Pa.s ($10^{4}$ Poise).

### 3.3. Relation between the Temperature and the Viscosity of Glass Melt

**Figure 3:**
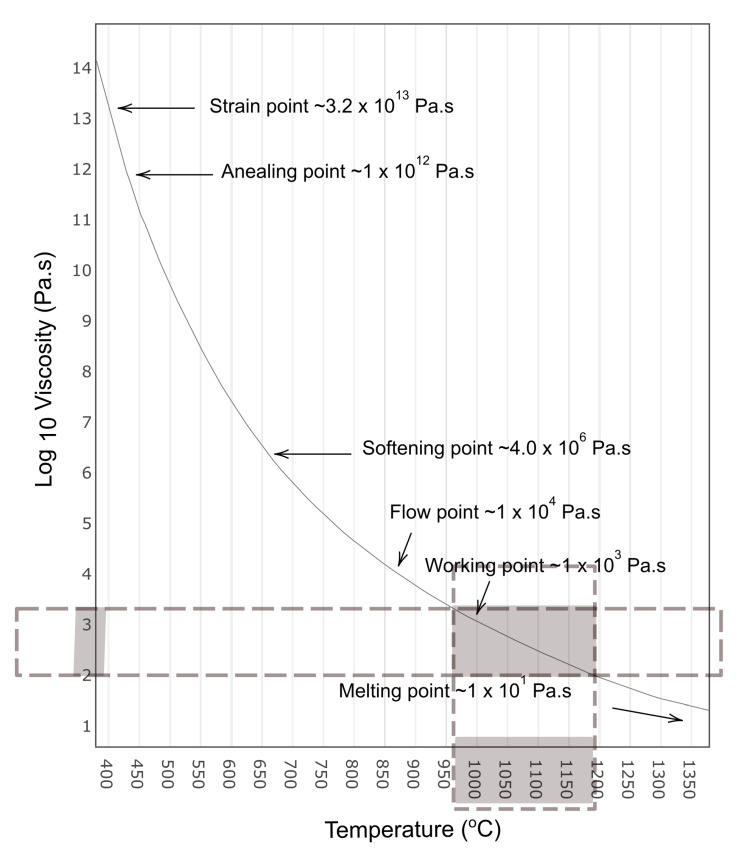
Viscosity temperature curve reconstructed from [7] with a highlighted region showing a nearly linear relationship between viscosity and temperature of the molten gob.

The viscosity temperature curve presented in Figure 3 is reconstructed from [7]. The highlighted part of the curve shows a relatively linear relationship between temperature and viscosity ranges that are vital to produce a good quality glass gob.

The gob temperature can be calculated with the Vogel–Tammann–Fulcher (VFT) equation from [28], i.e., (1).

(1) $$T=\frac{B}{log\eta +A}+T_{0}$$

where logη is the viscosities in log 10 base 10 scale, such as log2.0,log4.0 and log6.0, representing viscosities at different stages of glass melt. $T,T_{0}$ are temperatures in ${}^{\circ }$C; *A* and *B* are constant parameters, and *B* is activation energy for viscous flow.

## 4. Materials and Methods

### 4.1. Experimental Setup

**Figure 4:**
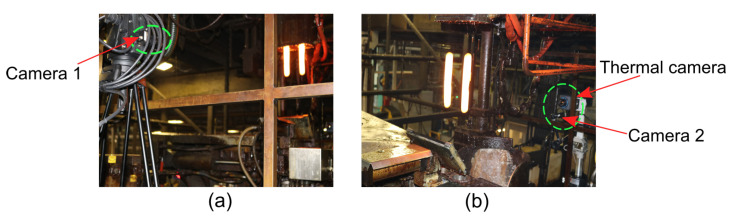
Multi-camera setup installed at a glass plant. (**a**) Thermal camera and camera 2. (**b**) Camera 1.

The measurement setup used in this article is similar to what was used in the article [17], with some modifications to adapt to the dimensions of the glass machine. The measurement setup was prepared for placement around an individual section IS machine [3,8] in a production line of a running glass plant. This multi-camera setup consists of two high-speed uEye UI-3060CP-M-GL-R2 monochrome cameras and an additional high-speed thermal camera FLIR A6752sc. The thermal camera was placed together with one of the high-speed cameras, as shown in Figure 4.

The cameras were equipped with 25 mm fixed focal length lenses. The selected frame rate for the high-speed cameras for this measurement was 377 frames per second with a cropped region of interest of 1936×500 pixels, and for the thermal camera, the frame rate was 125.6 frames per second. The exposure time for the high-speed cameras were set to 132 μs to minimize the effect of motion blur stated in [17].

**Figure 5:**
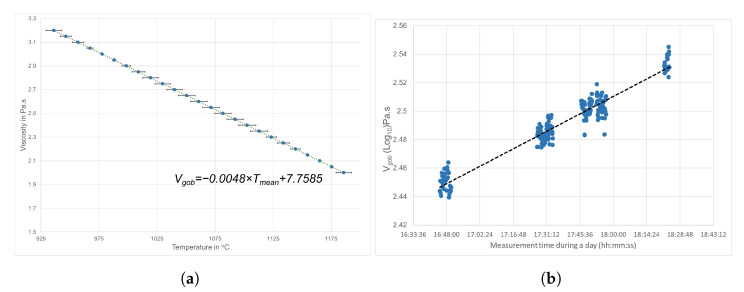
The plot in (**a**) shows the error and linear trend between the glass viscosity and temperature extracted from the highlighted part of Figure 3, and (**b**) shows the estimated $V_{gob}$.

The thermal camera operates in the 3-to-5 micron waveband. This waveband is appropriate to measure the gob temperature [29]. According to the data-sheet [30], for a moving object with a speed of 4 m/s, the motion blur will be 45% if the integration time is set to 2 ms. However, the measurements in this article used an integration time of 68 μs that not only reduces the motion blur, but also increases the frame rate up to 125 frames per second with a resolution of 640×512 pixels. The camera was tilted to 90 degrees to maximize the resolution of the vertical field of view (VFOV). The measurement error for this camera is ±2 ${}^{\circ }$C. The temperature data acquired from the camera is verified from Figure 5a as well as from [7,24,25,27]. From the literature it was found that ±2 ${}^{\circ }$C error in the measurement is acceptable since a considerable effect on glass viscosity would not be until the temperature variation is more than 20 ${}^{\circ }$C.

**Figure 6:**
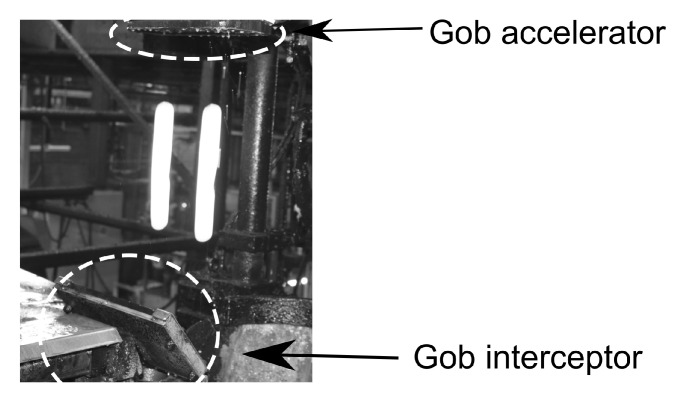
Gob interceptor and gob accelerator between the feeder and delivery system.

The working distance was set to 170 cm for maximum VFOV to cover the free-fall of the molten gob from feeder mechanism to delivery system. However, due to two additional machine components, as shown in Figure 6, called gob accelerator and gob interceptor located between the feeder and delivery mechanism, the effective VFOV where the free-falling gobs were visible was reduced to 54.7 cm from the intended 75.6 cm. Because of this issue, the resolution of the vertical field of view (VFOV) was reduced from 1936 pixels to 1401 pixels. Theoretically, the working distance could be increased, however, in this case, it was not possible because of the physical constraints around the production line. The gob accelerator is used to correct the alignment of molten gob after being cut from glass melt by a shear mechanism under the feeder mechanism. The gob interceptor is placed above the delivery system to discard the poorly produced gobs.

The cameras were mounted in the middle of the VFOV and parallel to the machine floor to avoid any tilt that may cause the gob to appear slightly bigger or smaller from either of the ends when free-falling between feeder and delivery system. According to the experiments conducted in [17], the slight deviation or variability in measured length is due to a segmentation error of about 0.16% and is low enough to be neglected.

**Figure 7:**
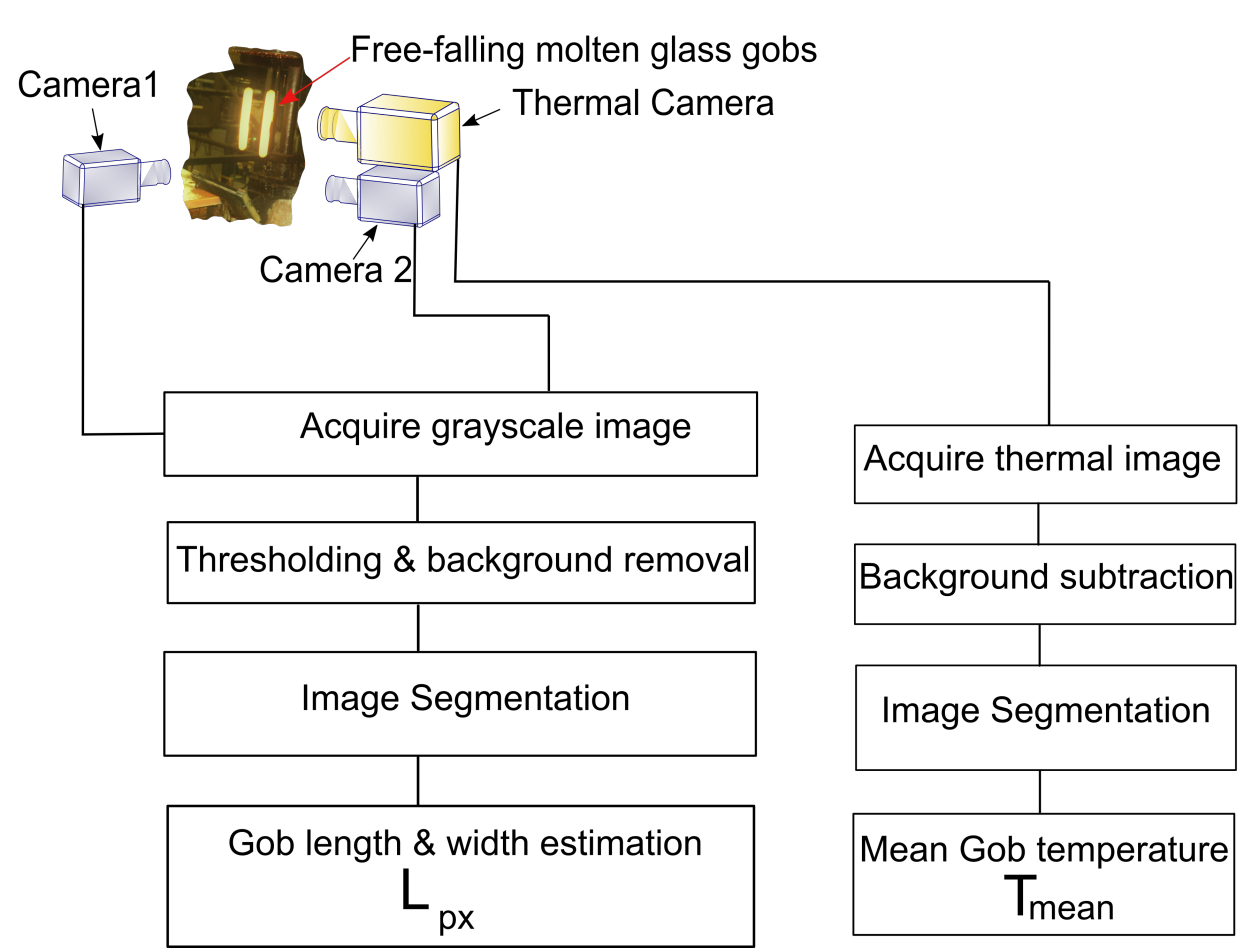
Gob capturing setup with two high-speed monochrome cameras and one high-speed thermal camera.

The block diagram in Figure 7 shows the thermal camera and two high-speed cameras. The captured images from the high-speed cameras were processed to calculate geometrical changes in the molten gob during free-fall. Geometrical changes in each falling position were calculated according to the method proposed in [17]. The thermal camera was used to acquire the thermal profile of molten gobs. Thermal images were captured and analyzed to extract the mean temperature of falling molten gobs.

To calibrate both high-speed cameras, and for the thermal camera to be able to capture the same gob, a small flash light was used in the beginning of each acquisition.

### 4.2. Methodology to Find the Relation between Gob Shape, Gob Temperature and Gob Viscosity

In order to calculate the geometrical changes in gob shape, captured images of free-falling molten gobs from high-speed cameras were analyzed. The length of the molten gobs was calculated with the method presented in [17].

#### 4.2.1. Determination of Geometrical Shape Change Rate, γ

During the free-fall of the molten gob between the gob accelerator and the gob interceptor, a change in its geometrical shape takes place. We denote the geometrical change rate as γ, and (2) is used to calculate γ for each molten gob.

(2) $$\gamma =\frac{\Delta L_{px}}{dt}$$

where $L_{px}$ is the length of the gob in pixels, dt is the change in time. Note that $L_{px}$ shows the length of the gob in pixels with both perspective and tilt corrections [17].

#### 4.2.2. Measurement of the Temperature of the Molten Gob

The glass composition was kept constant during the measurements. The composition used in this article was soda-lime-silica, which was based on [31]. The model presented in this article is mainly concerned with the viscosity range that is vital for the quality of the molten gob. According to the glass composition received from the glass plant and Table 1 and Figure 3, the range is $10^{2}$ Pa.s ($10^{3}$ Poise) to $10^{3.2}$ Pa.s ($10^{4.2}$ Poise).

In this study we analyzed the relation between γ and the viscosity of a molten gob relying on the dependence of viscosity to temperature variations. We omitted the chemical composition from the analysis, because it remains unchanged. The data in Figure 3 and Figure 5a suggest that the relationship between temperature and the viscosity of the molten gob is nearly linear. This motivates us to investigate the possible correlation between the changes in the geometrical shape of the molten gob and its viscosity. By showing a correlation between the temperature variation of the molten gob with its γ, we demonstrate that there is potential for a direct relation between viscosity and γ.

The images acquired from the thermal camera are used to estimate the gob temperature. The average temperature of a molten gob is calculated from a single frame by first subtracting the background using contour saliency [32] and then taking the arithmetic mean of the thermal profile of the extracted gob with (3) and (4). The raw pixel counts from the acquired thermal image of the gob is converted to units of temperature from the software provided by the thermal camera manufacturer. This temperature data is then further processed using (3) to subtract the background from the pixels representing gob temperature. Equation (4) is used to calculate the mean gob temperature $T_{mean}$. As pixels in each extracted gob represents temperature, in each frame where there is gob, the values are summed and divided by the number of pixels of the gob object.

(3) $$F=(I|p_{i,j}>th)$$

where *F* represents the image foreground with the gob object, including pixel values above a pre-selected threshold *th* for pixels belonging to a gob. Since the emissivity of molten glass is very high, i.e., closer to 0.98, the thermal image capturing software from FLIR ResearchIR, allows setting the emissivity value, which is helpful to acquire the threshold *th*. Due to the high emissivity of glass, the reflected temperature has less influence compared to lower emissivity objects where the reflected temperature from surrounding objects makes it difficult to measure the target. Therefore, the threshold *th* is the suggested value from the thermal image capturing software and it is based on the reflected value from the environmental temperature, while *I* is the thermal image.

The mean temperature $T_{mean}$ is derived as:

(4) $$T_{mean}=\frac{\sum_{All\;pixels}I\cap F}{\sum_{All\;pixels}F}$$

where *I* is the thermal image.

#### 4.2.3. Model to Correlate Viscosity and Temperature in the Molten Gob with Its γ

To find the relation between γ and gob viscosity we used the Pearson correlation coefficient (PCC) [33,34]. Equations (5) and (6) represent Pearson’s correlation coefficients $r_{1}$ and $r_{2}$, respectively, between γ and measurement temperature $T_{gob}$, and between γ and gob viscosity $V_{gob}$.

(5) $$r_{1}=\frac{\sum Z_{K}*Z_{T_{gob}}}{n-1}$$

(6) $$r_{2}=\frac{\sum Z_{K}*Z_{V_{gob}}}{n-1}$$

$Z_{K}$ can be calculated by taking each normalized value of γ, i.e., $\gamma_{norm}$, and subtracting that from the mean of $\gamma_{norm}$, i.e., $\bar{\gamma }$ and then dividing by the standard deviation of $\gamma_{norm}$, i.e., $\sigma_{K}$ as shown in (7)

(7) $$Z_{K}=\frac{\gamma_{norm}-\bar{\gamma }}{\sigma_{K}}$$

Similarly, the Z score for $Z_{T_{gob}}$ and $Z_{V_{gob}}$ can be calculated using (8) and (9)

(8) $$Z_{T_{gob}}=\frac{T_{gob}-\bar{T_{gob}}}{\sigma_{T_{gob}}}$$

(9) $$Z_{V_{gob}}=\frac{V_{gob}-\bar{V_{gob}}}{\sigma_{V_{gob}}}$$

where $T_{gob}$ is the normalized value of mean temperature of gob, $V_{gob}$ is the normalized value of estimated viscosity of the gob.

## 5. Results

**Figure 8:**
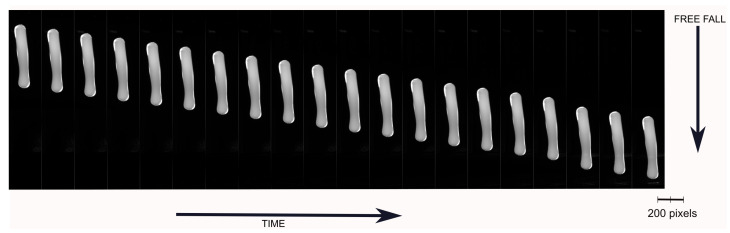
Sample images of free-falling molten gob.

**Figure 9:**
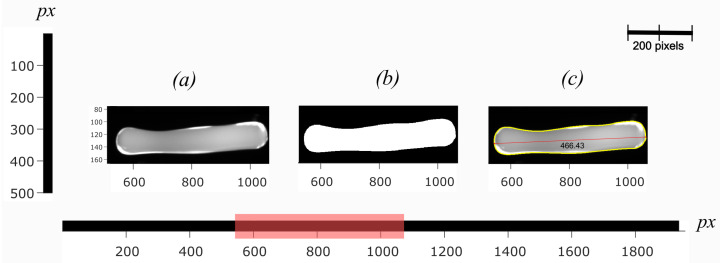
Measured length from the image of the molten gob taken using the setup presented in [17] (**a**) original image of the gob, (**b**) binary image of the gob, (**c**) original image with boundaries and length ($L_{px}$) [17].

Figure 8 shows the acquired images of a free-falling molten gob between the feeder mechanism and delivery system. The processing steps to estimate measured length in pixels ($L_{px}$) from the captured gob images are presented in Figure 9.

**Figure 10:**
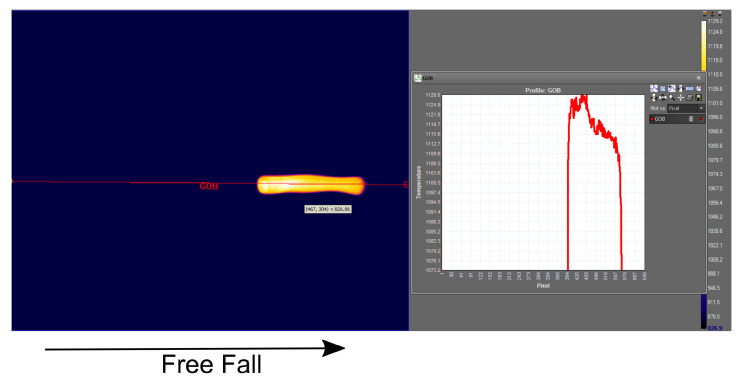
Thermal image of a falling gob shows gob temperature during free fall.

**Figure 11:**
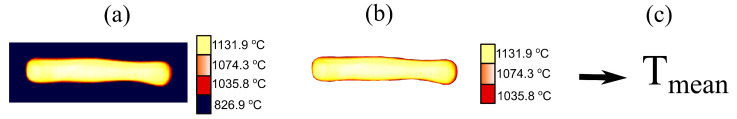
Thermal image processing steps to calculate mean gob temperature $T_{mean}$ of the molten gob (**a**) original thermal image of the gob, (**b**) thermal image after background subtraction and image segmentation, and (**c**) $T_{mean}$ of the molten gob.

Figure 10 shows a thermal image of a free falling gob with its profile temperature. The threshold *th* in (3) to subtract background is determined through the mean of the surrounding temperature. Figure 11 shows the processing steps to calculate mean gob temperature $T_{mean}$ from thermal image of a molten gob.

**Figure 12:**
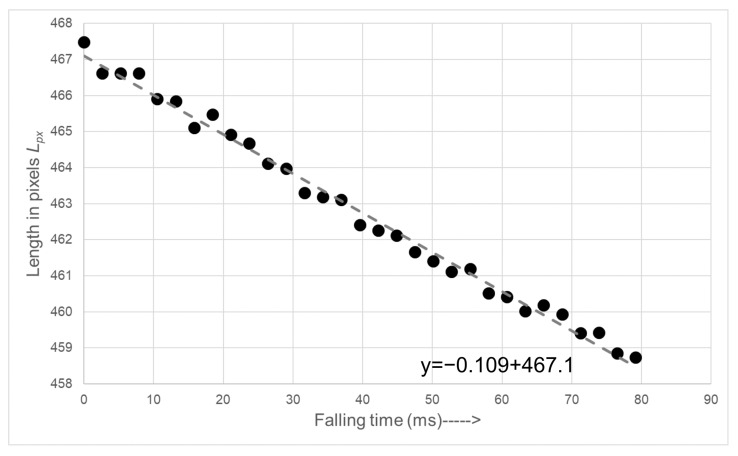
Gob length in pixels vs. time in free fall.

The plot in Figure 12 shows the length vs. time of the free-falling molten gob measured in number of pixels. Moreover, we calculated the γ of each gob using (2), where every γ represents the deformation of a given gob. The collected γ for several gob samples are then plotted against the total number of frames $f_{gob}$ that each molten gob took during its free fall from fore-hearth to delivery system.

**Figure 13:**
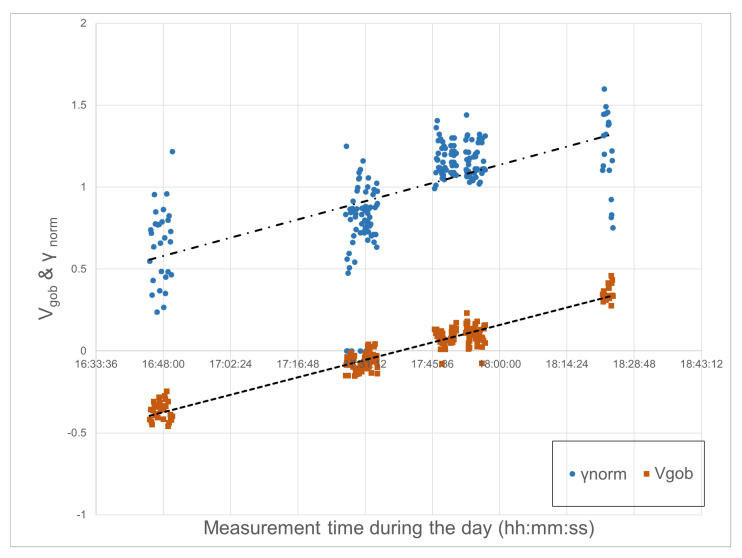
$\gamma_{norm}$ and normalized estimated gob viscosity values plotted against measuring events in time.

Linear regression is used to calculate the normalized γ using the number of frames and γ. A $\gamma_{norm}$ can be achieved by dividing each γ with values extracted from linear regression. The $\gamma_{norm}$ is then plotted against the measurement time as shown in Figure 13.

In this experiment, we observed that there is an inverse linear relationship between the shape change of the molten gob γ and the measured mean temperature of the molten gob. Therefore, the estimated viscosity from the measured gob temperature can be extrapolated by using data from Figure 3 through linear regression, as shown in Figure 5b.

## 6. Discussion

The method introduced in this article relies on two high-speed cameras for capturing the geometrical shape changes of free-falling gob and a high-speed thermal camera for verifying the temperature profile of the falling gob. An important consideration in this setup was made for synchronization of the cameras to capture and identify the same gob. In this case, the glass gobs are sequential with falling periods in milliseconds, the difference in frame rate in the high-speed camera and thermal camera does not generate any jitter problems, since one value per gob and video stream is generated. Thus, each value is paired with a specific gob, which makes their detection and identification simple with the chosen high-speed cameras and the thermal camera. Another element that required consideration in our analysis is the accuracy of the measurements with respect to the environmental changes around the glass forming machine. Even though glass is considered as isothermal [35] from the inside, the surface temperature changes quickly after the gob has been produced. Therefore, accurate measurements of surface temperature are important to minimize measurement error. During this experiment, humidity and the surrounding temperature have been constantly monitored to ensure the accuracy of the temperature measurements. For further validation, the measured temperature is compared with the typical gob temperature ranges mentioned in Figure 5a. However, the uncertainty associated with this measurement setup comes from three main sources: (1) length measurement, (2) temperature measurement and (3) variation in the chemical composition of glass. The first source or uncertainty is due to a segmentation error while capturing images from the high speed camera, which is about 0.16% [17]. The second source or uncertainty is due to determining the temperature, which is about ±2 ${}^{\circ }$C according to the thermal camera specification, and the third is the glass composition that can also be a source of error. For example, impurities and unknown compositions may slightly vary the temperature viscosity relationship. As the glass melt is mixed during production, we consider this uncertainty to be very low. Thus, the combined uncertainty of the measurement is smaller than the spread of the data points, which means that the uncertainty will not affect the conclusion of this work.

The calculated Pearson correlation coefficient $r_{1}$ for γ of a gob and the mean temperature of the gob is −0.65, which suggests an inverse linear relationship between gob temperature and γ. In contrast, the calculated Pearson correlation coefficient $r_{2}$ for γ of gob and estimated viscosity of gob as shown in Figure 13 is 0.65, which suggests a direct linear relationship between the two. According to the guides suggested by Evans [36], the Pearson correlation coefficient is considered to be strong if the value is between 0.60–0.79.

In this article, we found a linear correlation between γ of a gob and its viscosity. Since viscosity is a relatively slow process, many gobs can be measured to reduce the error for one measurement point. This motivates research in validating this method into a contactless tool for early detection of compromise in gob quality.

Viscosity is dependent on temperature and the chemical composition of the glass melt. The results in this paper are captured during a single glass melt, which means that the temperature directly correlates to the temperature of the glass for these measurements. Thus, the thermal camera and measurement of gob temperature enable the correlation analysis between the proposed method to estimate viscosity with temperature. The thermal camera in Figure 7 enables correlation between the geometrical shape change, γ, and the viscosity. This is possible since it gives a temperature to estimate the relative change in viscosity that is bound to a certain glass composition. However, viscosity is not just dependent on temperature, it also depends on glass composition, therefore, the use of only a thermal camera would give a temperature for the estimation of relative change in viscosity that is bound to a certain glass composition. However, we know from [24] and [25] that there is a linear relationship between the surface tension and temperature of molten glass, therefore, it is possible to use two off-the-shelf high speed cameras for a fraction of the cost of a thermal camera to estimate changes in the viscosity of the molten gob by calculating γ of molten gobs; such measurements provide a viscosity estimation for different glass compositions. However, in this article, a soda-lime-silica glass composition is used, and further research is needed to validate this method for other glass compositions. This work opens a potential for further investigation into in-line measurement systems by including more glass compositions in the analysis to develop a robust tool, which could be useful for early fault detection in the glass container production process.

## 7. Conclusions

In this article, we show that there is a correlation between the geometrical shape changes of a free-falling glass gob to its viscosity. The calculated correlation coefficient is 0.65, which indicates a strong correlation between the two. The geometrical change and glass gob temperature is captured using two high-speed cameras and one thermal camera. From these measurements, the correlation models presented in this article showed that it is possible to use this approach to measure the relative viscosity of molten gobs in real-time. To get absolute viscosity, the combination of geometrical change and gob temperature needs to be analyzed to estimate absolute viscosity in a controlled environment, where the temperature and glass melt can be controlled independently. An in-line measurement that can detect variations can enable a closed-loop control in an early production stage of glass containers. For example, measurement of viscosity can improve the overall control of the glass melt, consequently minimizing error in the glass formation by optimizing the viscosity in real-time. Thus, it would improve glass container production quality and efficiency.

## Figures and Tables

**Table 1:** Viscosity ranges for glass container manufacturing [27].

Viscosity Ranges from some Glass Manufacturing Operations.	
*Note: viscosity values are converted to log_10_ Pa.s*	
Glass melting	0.5 to 1.5
Sealing glasses to other glasses or to metals	2.5 to 2.8
Producing gobs for container forming	2.6 to 3.2
Drawing sheet glass (Fourcault process)	3.0 to 3.4
Glass pressing	3.0 to 5.3
Surface of a bottle during blowing	4.7 to 9.0
Sinter glass powder to a solid body	5.0
Sinter glass powder to form a porous body	7.0 to 7.8
Dilatometric softening point	10.3 to 10.7
Annealing range	11.0 to 13.0
Stress release occurs in a few seconds	11.8
Temperature for matching expansion curves for seals	13.0 to 13.5
Stress release too slow to be useful	above 13.6

## Data Availability

Not applicable.
